# Supplementary material for: Venomix: a simple bioinformatic pipeline for identifying and characterizing toxin gene candidates from transcriptomic data
Source: PeerJ. 2018 Jul 31;6:e5361. doi: 10.7717/peerj.5361 (PMC6074769; doi:10.7717/peerj.5361)
Supplement: Supplemental Information 6 [file peerj-06-5361-s006.gz › FinalOutput_1E-6/Thrombin-like_enzyme_ancrod_11/finaltree.pdf]

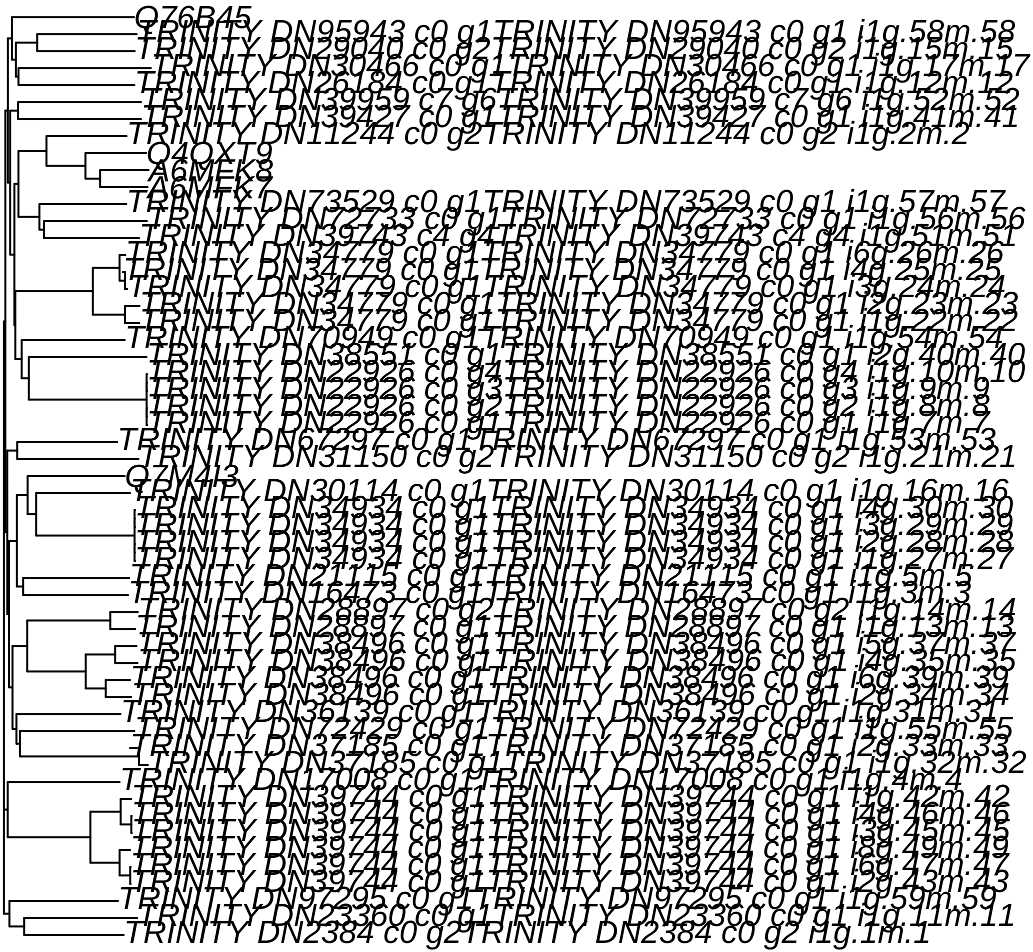
Q76B45
TRINITY DN95943 c0 g1TRINITY DN95943 c0 g1 i1g.58m.58
TRINITY DN29040 c0 g2TRINITY DN29040 c0 g2 i1g.15m.15
TRINITY DN30466 c0 g1TRINITY DN30466 c0 g1 i1g.17m.17
TRINITY DN26184 c0 g1TRINITY DN26184 c0 g1 i1g.12m.12
TRINITY DN39959 c7 g6TRINITY DN39959 c7 g6 i1g.52m.52
TRINITY DN39427 c0 g1TRINITY DN39427 c0 g1 i1g.41m.41
TRINITY DN11244 c0 g2TRINITY DN11244 c0 g2 i1g.2m.2
Q4QXT9
A6MEK8
A6MEK7
TRINITY DN73529 c0 g1TRINITY DN73529 c0 g1 i1g.57m.57
TRINITY DN72733 c0 g1TRINITY DN72733 c0 g1 i1g.56m.56
TRINITY DN39743 c4 g4TRINITY DN39743 c4 g4 i1g.51m.51
TRINITY DN34779 c0 g1TRINITY DN34779 c0 g1 i6g.26m.26
TRINITY DN34779 c0 g1TRINITY DN34779 c0 g1 i4g.25m.25
TRINITY DN34779 c0 g1TRINITY DN34779 c0 g1 i3g.24m.24
TRINITY DN34779 c0 g1TRINITY DN34779 c0 g1 i2g.23m.23
TRINITY DN34779 c0 g1TRINITY DN34779 c0 g1 i1g.22m.22
TRINITY DN70949 c0 g1TRINITY DN70949 c0 g1 i1g.54m.54
TRINITY DN38551 c0 g1TRINITY DN38551 c0 g1 i2g.40m.40
TRINITY DN22926 c0 g4TRINITY DN22926 c0 g4 i1g.10m.10
TRINITY DN22926 c0 g3TRINITY DN22926 c0 g3 i1g.9m.9
TRINITY DN22926 c0 g2TRINITY DN22926 c0 g2 i1g.8m.8
TRINITY DN22926 c0 g1TRINITY DN22926 c0 g1 i1g.7m.7
TRINITY DN67297 c0 g1TRINITY DN67297 c0 g1 i1g.53m.53
TRINITY DN31150 c0 g2TRINITY DN31150 c0 g2 i1g.21m.21
Q7M4I3
TRINITY DN30114 c0 g1TRINITY DN30114 c0 g1 i1g.16m.16
TRINITY DN34934 c0 g1TRINITY DN34934 c0 g1 i4g.30m.30
TRINITY DN34934 c0 g1TRINITY DN34934 c0 g1 i3g.29m.29
TRINITY DN34934 c0 g1TRINITY DN34934 c0 g1 i2g.28m.28
TRINITY DN34934 c0 g1TRINITY DN34934 c0 g1 i1g.27m.27
TRINITY DN21115 c0 g1TRINITY DN21115 c0 g1 i1g.5m.5
TRINITY DN16473 c0 g1TRINITY DN16473 c0 g1 i1g.3m.3
TRINITY DN28897 c0 g2TRINITY DN28897 c0 g2 i1g.14m.14
TRINITY DN28897 c0 g1TRINITY DN28897 c0 g1 i1g.13m.13
TRINITY DN38496 c0 g1TRINITY DN38496 c0 g1 i5g.37m.37
TRINITY DN38496 c0 g1TRINITY DN38496 c0 g1 i4g.35m.35
TRINITY DN38496 c0 g1TRINITY DN38496 c0 g1 i6g.39m.39
TRINITY DN38496 c0 g1TRINITY DN38496 c0 g1 i2g.34m.34
TRINITY DN36139 c0 g1TRINITY DN36139 c0 g1 i1g.31m.31
TRINITY DN72429 c0 g1TRINITY DN72429 c0 g1 i1g.55m.55
TRINITY DN37185 c0 g1TRINITY DN37185 c0 g1 i2g.33m.33
TRINITY DN37185 c0 g1TRINITY DN37185 c0 g1 i1g.32m.32
TRINITY DN17008 c0 g1TRINITY DN17008 c0 g1 i1g.4m.4
TRINITY DN39744 c0 g1TRINITY DN39744 c0 g1 i1g.42m.42
TRINITY DN39744 c0 g1TRINITY DN39744 c0 g1 i4g.46m.46
TRINITY DN39744 c0 g1TRINITY DN39744 c0 g1 i3g.45m.45
TRINITY DN39744 c0 g1TRINITY DN39744 c0 g1 i8g.49m.49
TRINITY DN39744 c0 g1TRINITY DN39744 c0 g1 i6g.47m.47
TRINITY DN39744 c0 g1TRINITY DN39744 c0 g1 i2g.43m.43
TRINITY DN97295 c0 g1TRINITY DN97295 c0 g1 i1g.59m.59
TRINITY DN23360 c0 g1TRINITY DN23360 c0 g1 i1g.11m.11
TRINITY DN2384 c0 g2TRINITY DN2384 c0 g2 i1g.1m.1
